# Bis(triphenyl-λ^5^-phosphanylidene)ammonium hydrogen dichloride

**DOI:** 10.1107/S1600536811035057

**Published:** 2011-09-03

**Authors:** Jorit Gellhaar, Carsten Knapp

**Affiliations:** a Institut für Anorganische und Analytische Chemie, Albert-Ludwigs-Universität Freiburg, Albertstrasse 21, 79104 Freiburg i. Br., Germany

## Abstract

In the title compound, [(Ph_3_P)_2_N]^+^·[Cl-H-Cl]^−^ or C_36_H_30_NP_2_^+^·Cl_2_H^−^, the H atom of the [Cl—H—Cl]^−^ anion and the N atom of the [(Ph_3_P)_2_N]^+^ cation are located on a twofold axis, yielding overall symmetry 2 for both the cation and the anion. The central P—N—P angle [144.12 (13)°] of the cation is in the expected range and indicates only weak cation–anion inter­actions. The almost linear [Cl—H—Cl]^−^ anion is a rare example of a symmetric hydrogen bridge in a hydrogen dichloride anion. The Cl⋯Cl distance and two equal Cl—H bonds are typical of such a symmetric hydrogen dichloride anion.

## Related literature

For selected examples containing the [Cl—H—Cl]^−^ anion, see: Atwood *et al.* (1990 ▶); Mootz *et al.* (1981 ▶); Habtemariam *et al.* (2001 ▶); Swann *et al.* (1984 ▶); Neumüller *et al.* (2005 ▶). For other bis(triphenyl-λ^5^-phosphanylidene)ammonium halide structures, see: Knapp & Uzun (2010**a* ▶,b* ▶); Beckett *et al.* (2010 ▶). For a discussion of the [(Ph_3_P)_2_N]^+^ cation, see: Lewis & Dance (2000 ▶). For a description of the Cambridge Structural Database, see: Allen (2002 ▶). For the synthesis of [(Ph_3_P)_2_N]Cl, see: Ruff & Schlientz (1974 ▶).
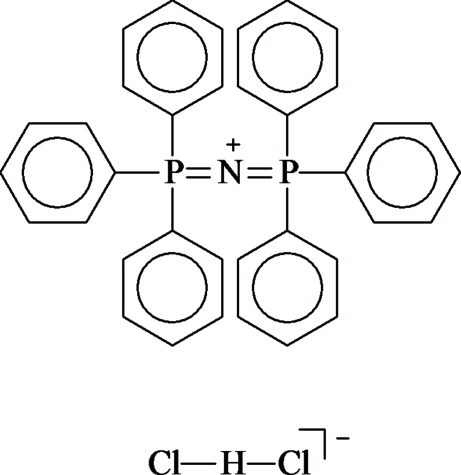


## Experimental

### 

#### Crystal data

C_36_H_30_NP_2_^+^·Cl_2_H^−^
*M* *_r_* = 610.46
Orthorhombic, 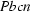

*a* = 11.6467 (3) Å
*b* = 16.5474 (4) Å
*c* = 15.7584 (3) Å
*V* = 3037.00 (12) Å^3^
*Z* = 4
Mo *K*α radiation
μ = 0.35 mm^−1^
*T* = 100 K
0.18 × 0.14 × 0.09 mm

#### Data collection

Bruker APEXII CCD area-detector diffractometer
Absorption correction: multi-scan (*SADABS*; Bruker, 2001 ▶) *T*_min_ = 0.940, *T*_max_ = 0.970
28633 measured reflections
2994 independent reflections
2671 reflections with *I* > 2σ(*I*)
*R*_int_ = 0.030

#### Refinement

*R*[*F* ^2^ > 2σ(*F* ^2^)] = 0.033
*wR*(*F* ^2^) = 0.085
*S* = 1.08
2994 reflections
189 parameters
H atoms treated by a mixture of independent and constrained refinement
Δρ_max_ = 0.61 e Å^−3^
Δρ_min_ = −0.79 e Å^−3^

### 

Data collection: *APEX2* (Bruker, 2007 ▶); cell refinement: *SAINT* (Bruker, 2007 ▶); data reduction: *SAINT*; program(s) used to solve structure: *SHELXS97* (Sheldrick, 2008 ▶); program(s) used to refine structure: *SHELXL97* (Sheldrick, 2008 ▶); molecular graphics: *DIAMOND* (Brandenburg & Putz, 2011 ▶); software used to prepare material for publication: *SHELXL97*.

## Supplementary Material

Crystal structure: contains datablock(s) I, global. DOI: 10.1107/S1600536811035057/su2304sup1.cif

Structure factors: contains datablock(s) I. DOI: 10.1107/S1600536811035057/su2304Isup2.hkl

Additional supplementary materials:  crystallographic information; 3D view; checkCIF report

## Figures and Tables

**Table 1:** Hydrogen-bond geometry (Å, °)

*D*—H⋯*A*	*D*—H	H⋯*A*	*D*⋯*A*	*D*—H⋯*A*
Cl1—H1⋯Cl1^i^	1.56 (1)	1.56 (1)	3.1045 (9)	173 (3)

Symmetry code: (i) 
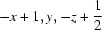
.

**Fig. 1.**
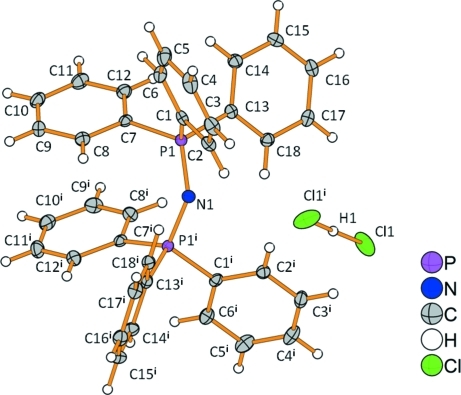
A view of the ionic unit of the title compound, showing the atom numbering and displacement ellipsoids drawn at the 50% probability level. H atoms are drawn with arbitrary radii. [Symmetry code: (i) -x+1, y, -z+0.5.]

## supplementary crystallographic information

### Comment

The Cambridge Structural Database (Allen, 2002) currently contains more than 1200 structures containing the [(Ph_3_P)_2_N]^+^ cation ([(Ph_3_P)_2_N]^+^ = [PNP]^+^). Usually this cation is partnered by a bulky anion, while crystal structures containing small anions and especially halides are rare. The crystal structures of some solvate-free halides, [(Ph_3_P)_2_N]I (Beckett *et al.*, 2010) and [(Ph_3_P)_2_N]Cl (Knapp & Uzun, 2010*a*), and the acetontrile solvate [(Ph_3_P)_2_N]Br^.^CH_3_CN (Knapp & Uzun, 2010*b*), were published only very recently.

Bis(triphenyl-λ^5^-phosphanylidene)ammonium chloride [(Ph_3_P)_2_N]Cl was synthesized according to a published method (Ruff *et al.*, 1974). Solvate-free single crystals suitable for X-ray diffraction of the title compound were obtained as a by-product by layering a CH_3_CN solution with diethyl ether. The H atom of the [Cl—H—Cl]^-^ anion and the N atom of the [(Ph_3_P)_2_N]^+^ cation are located on a twofold axis, yielding overall symmetry 2 for the cation. The central P—N—P angle [144.12 (13)°] and the P—N [1.5762 (7) Å] and P—C distances [1.7940 (16)–1.8028 (15) Å] are in the expected range for the [(Ph_3_P)_2_N]^+^ cation (Lewis & Dance, 2000).

The number of structurally characterized hydrogen dichloride anions is still limited. Often disorder or low crystal quality does not allow to establish unequivically the position of the H atom. The Cl···Cl distances in all hydrogen dichloride anions deviate only slightly from an averaged distance of 3.10 Å. The anion in the title compound contains a symmetric hydrogen bridge with H—Cl distances of 1.56 (1) Å and a Cl···Cl distance of 3.1045 (9) Å. The anion is almost linear and a Cl—H—Cl angle of 173 (3)° is observed. A very similar hydrogen dichloride anion was found in the the crystal structure of [K(18 C-6)][Cl—H—Cl], where H—Cl is 1.56 Å and Cl···Cl 3.117 (1) Å (Atwood *et al.*, 1990).

### Experimental

Single crystals of the title compound, suitable for X-ray diffraction analysis, were obtained as a by-product by layering a CH_3_CN solution of [(Ph_3_P)_2_N]Cl with diethyl ether.

### Refinement

The carbon-bonded hydrogen atoms were positioned geometrically and refined using a riding model. The same *U_iso_* value was used for all H atoms, which refined to 0.0226 (13) Å^2^. The coordinates for the hydrogen atom in the [Cl—H—Cl]^-^ anion were taken from the Fourier map and the atom was refined isotropically.

### Crystal data

**Table:**

C_36_H_30_NP_2_^+^·Cl_2_H^−^	*F*(000) = 1272
*M**_r_* = 610.46	*D*_x_ = 1.335 Mg m^−^^3^
Orthorhombic, *P**b**c**n*	Mo *K*α radiation, λ = 0.71073 Å
Hall symbol: -P 2n 2ab	Cell parameters from 9956 reflections
*a* = 11.6467 (3) Å	θ = 2.5–30.8°
*b* = 16.5474 (4) Å	µ = 0.35 mm^−^^1^
*c* = 15.7584 (3) Å	*T* = 100 K
*V* = 3037.00 (12) Å^3^	Block, colourless
*Z* = 4	0.18 × 0.14 × 0.09 mm

### Data collection

**Table:**

Bruker APEXII CCD area-detector diffractometer	2994 independent reflections
Radiation source: microfocus sealed tube	2671 reflections with *I* > 2σ(*I*)
multilayer mirro optics	*R*_int_ = 0.030
φ and ω scans	θ_max_ = 26.0°, θ_min_ = 2.1°
Absorption correction: multi-scan (*SADABS*; Bruker, 2001)	*h* = −14→14
*T*_min_ = 0.940, *T*_max_ = 0.970	*k* = −19→20
28633 measured reflections	*l* = −19→16

### Refinement

**Table:**

Refinement on *F*^2^	Primary atom site location: structure-invariant direct methods
Least-squares matrix: full	Secondary atom site location: difference Fourier map
*R*[*F*^2^ > 2σ(*F*^2^)] = 0.033	Hydrogen site location: inferred from neighbouring sites
*wR*(*F*^2^) = 0.085	H atoms treated by a mixture of independent and constrained refinement
*S* = 1.08	*w* = 1/[σ^2^(*F*_o_^2^) + (0.0372*P*)^2^ + 2.5322*P*] where *P* = (*F*_o_^2^ + 2*F*_c_^2^)/3
2994 reflections	(Δ/σ)_max_ < 0.001
189 parameters	Δρ_max_ = 0.61 e Å^−^^3^
0 restraints	Δρ_min_ = −0.79 e Å^−^^3^

### Special details

**Table:**

Geometry. All e.s.d.'s (except the e.s.d. in the dihedral angle between two l.s. planes) are estimated using the full covariance matrix. The cell e.s.d.'s are taken into account individually in the estimation of e.s.d.'s in distances, angles and torsion angles; correlations between e.s.d.'s in cell parameters are only used when they are defined by crystal symmetry. An approximate (isotropic) treatment of cell e.s.d.'s is used for estimating e.s.d.'s involving l.s. planes.
Refinement. Refinement of *F*^2^ against ALL reflections. The weighted *R*-factor *wR* and goodness of fit *S* are based on *F*^2^, conventional *R*-factors *R* are based on *F*, with *F* set to zero for negative *F*^2^. The threshold expression of *F*^2^ > σ(*F*^2^) is used only for calculating *R*-factors(gt) *etc*. and is not relevant to the choice of reflections for refinement. *R*-factors based on *F*^2^ are statistically about twice as large as those based on *F*, and *R*- factors based on ALL data will be even larger.

### Fractional atomic coordinates and isotropic or equivalent isotropic displacement parameters (Å^2^)

**Table:**

	*x*	*y*	*z*	*U*_iso_*/*U*_eq_	
P1	0.37492 (3)	0.47726 (2)	0.22743 (2)	0.01086 (11)	
N1	0.5000	0.44792 (11)	0.2500	0.0142 (4)	
C1	0.32847 (13)	0.42600 (9)	0.13289 (10)	0.0136 (3)	
C2	0.37451 (13)	0.35003 (10)	0.11482 (10)	0.0163 (3)	
H2	0.4320	0.3275	0.1505	0.0226 (13)*	
C3	0.33594 (15)	0.30750 (10)	0.04441 (11)	0.0203 (4)	
H3	0.3678	0.2561	0.0315	0.0226 (13)*	
C4	0.25109 (16)	0.33988 (10)	−0.00703 (11)	0.0227 (4)	
H4	0.2247	0.3104	−0.0549	0.0226 (13)*	
C5	0.20431 (15)	0.41512 (11)	0.01091 (11)	0.0224 (4)	
H5	0.1460	0.4369	−0.0245	0.0226 (13)*	
C6	0.24288 (14)	0.45863 (10)	0.08092 (10)	0.0173 (3)	
H6	0.2112	0.5102	0.0933	0.0226 (13)*	
C7	0.36204 (12)	0.58392 (9)	0.20936 (10)	0.0126 (3)	
C8	0.43189 (13)	0.61829 (10)	0.14660 (10)	0.0171 (3)	
H8	0.4784	0.5847	0.1118	0.0226 (13)*	
C9	0.43306 (14)	0.70100 (10)	0.13533 (11)	0.0203 (4)	
H9	0.4809	0.7243	0.0931	0.0226 (13)*	
C10	0.36455 (14)	0.75028 (10)	0.18551 (11)	0.0204 (4)	
H10	0.3661	0.8072	0.1778	0.0226 (13)*	
C11	0.29393 (15)	0.71670 (10)	0.24675 (11)	0.0210 (4)	
H11	0.2462	0.7505	0.2803	0.0226 (13)*	
C12	0.29273 (14)	0.63361 (10)	0.25912 (10)	0.0169 (3)	
H12	0.2447	0.6107	0.3015	0.0226 (13)*	
C13	0.27637 (13)	0.44720 (9)	0.30980 (10)	0.0128 (3)	
C14	0.15825 (13)	0.45901 (10)	0.30003 (10)	0.0162 (3)	
H14	0.1295	0.4861	0.2513	0.0226 (13)*	
C15	0.08307 (14)	0.43109 (10)	0.36169 (11)	0.0189 (3)	
H15	0.0028	0.4391	0.3550	0.0226 (13)*	
C16	0.12438 (14)	0.39159 (10)	0.43294 (10)	0.0179 (3)	
H16	0.0724	0.3723	0.4748	0.0226 (13)*	
C17	0.24172 (14)	0.38023 (10)	0.44327 (10)	0.0174 (3)	
H17	0.2700	0.3534	0.4923	0.0226 (13)*	
C18	0.31776 (13)	0.40806 (9)	0.38184 (10)	0.0149 (3)	
H18	0.3980	0.4004	0.3890	0.0226 (13)*	
Cl1	0.49306 (4)	0.14372 (3)	0.15163 (3)	0.03665 (15)	
H1	0.5000	0.149 (3)	0.2500	0.087 (15)*	

### Atomic displacement parameters (Å^2^)

**Table:**

	*U*^11^	*U*^22^	*U*^33^	*U*^12^	*U*^13^	*U*^23^
P1	0.0108 (2)	0.0097 (2)	0.0120 (2)	−0.00044 (14)	−0.00017 (14)	−0.00049 (14)
N1	0.0123 (9)	0.0108 (9)	0.0196 (10)	0.000	−0.0001 (7)	0.000
C1	0.0144 (7)	0.0138 (8)	0.0127 (7)	−0.0053 (6)	0.0026 (6)	−0.0011 (6)
C2	0.0148 (7)	0.0154 (8)	0.0188 (8)	−0.0027 (6)	0.0026 (6)	−0.0021 (6)
C3	0.0231 (8)	0.0158 (8)	0.0219 (9)	−0.0040 (7)	0.0065 (7)	−0.0059 (7)
C4	0.0334 (9)	0.0200 (9)	0.0146 (8)	−0.0099 (7)	0.0004 (7)	−0.0030 (7)
C5	0.0300 (9)	0.0212 (9)	0.0160 (8)	−0.0063 (7)	−0.0066 (7)	0.0041 (7)
C6	0.0229 (8)	0.0135 (8)	0.0155 (8)	−0.0021 (6)	−0.0011 (6)	0.0015 (6)
C7	0.0126 (7)	0.0118 (7)	0.0134 (7)	0.0000 (6)	−0.0044 (6)	−0.0001 (6)
C8	0.0160 (7)	0.0169 (8)	0.0184 (8)	0.0015 (6)	0.0007 (6)	0.0006 (6)
C9	0.0187 (8)	0.0174 (9)	0.0247 (9)	−0.0021 (7)	−0.0007 (7)	0.0067 (7)
C10	0.0218 (8)	0.0118 (8)	0.0276 (9)	0.0007 (6)	−0.0076 (7)	0.0019 (7)
C11	0.0231 (8)	0.0158 (8)	0.0240 (9)	0.0054 (7)	−0.0021 (7)	−0.0027 (7)
C12	0.0173 (8)	0.0166 (8)	0.0169 (8)	0.0019 (6)	0.0003 (6)	0.0001 (6)
C13	0.0148 (7)	0.0106 (7)	0.0130 (8)	−0.0014 (6)	0.0006 (6)	−0.0023 (6)
C14	0.0155 (7)	0.0185 (8)	0.0147 (8)	−0.0014 (6)	−0.0018 (6)	0.0002 (6)
C15	0.0133 (7)	0.0214 (9)	0.0219 (9)	−0.0024 (6)	0.0001 (6)	−0.0006 (7)
C16	0.0202 (8)	0.0153 (8)	0.0180 (8)	−0.0039 (6)	0.0047 (6)	0.0001 (6)
C17	0.0230 (8)	0.0142 (8)	0.0149 (8)	0.0002 (6)	−0.0009 (6)	0.0022 (6)
C18	0.0151 (7)	0.0127 (8)	0.0168 (8)	0.0008 (6)	−0.0009 (6)	−0.0005 (6)
Cl1	0.0363 (3)	0.0556 (3)	0.0181 (2)	0.0236 (2)	−0.00128 (18)	−0.0032 (2)

### Geometric parameters (Å, °)

**Table:**

P1—N1	1.5762 (7)	C9—C10	1.388 (2)
P1—C7	1.7940 (16)	C9—H9	0.9500
P1—C1	1.7976 (16)	C10—C11	1.384 (2)
P1—C13	1.8028 (15)	C10—H10	0.9500
N1—P1^i^	1.5761 (7)	C11—C12	1.389 (2)
C1—C2	1.396 (2)	C11—H11	0.9500
C1—C6	1.399 (2)	C12—H12	0.9500
C2—C3	1.389 (2)	C13—C18	1.393 (2)
C2—H2	0.9500	C13—C14	1.398 (2)
C3—C4	1.386 (3)	C14—C15	1.387 (2)
C3—H3	0.9500	C14—H14	0.9500
C4—C5	1.388 (3)	C15—C16	1.386 (2)
C4—H4	0.9500	C15—H15	0.9500
C5—C6	1.392 (2)	C16—C17	1.389 (2)
C5—H5	0.9500	C16—H16	0.9500
C6—H6	0.9500	C17—C18	1.390 (2)
C7—C12	1.394 (2)	C17—H17	0.9500
C7—C8	1.401 (2)	C18—H18	0.9500
C8—C9	1.380 (2)	Cl1—H1	1.555 (3)
C8—H8	0.9500		
			
N1—P1—C7	114.59 (8)	C8—C9—C10	120.24 (16)
N1—P1—C1	108.65 (7)	C8—C9—H9	119.9
C7—P1—C1	107.91 (7)	C10—C9—H9	119.9
N1—P1—C13	109.93 (6)	C11—C10—C9	120.19 (15)
C7—P1—C13	109.42 (7)	C11—C10—H10	119.9
C1—P1—C13	105.96 (7)	C9—C10—H10	119.9
P1^i^—N1—P1	144.12 (13)	C10—C11—C12	120.08 (16)
C2—C1—C6	120.13 (14)	C10—C11—H11	120.0
C2—C1—P1	118.58 (12)	C12—C11—H11	120.0
C6—C1—P1	121.17 (12)	C11—C12—C7	119.95 (16)
C3—C2—C1	119.67 (15)	C11—C12—H12	120.0
C3—C2—H2	120.2	C7—C12—H12	120.0
C1—C2—H2	120.2	C18—C13—C14	119.68 (14)
C4—C3—C2	120.14 (16)	C18—C13—P1	119.66 (11)
C4—C3—H3	119.9	C14—C13—P1	120.57 (12)
C2—C3—H3	119.9	C15—C14—C13	119.84 (15)
C3—C4—C5	120.49 (16)	C15—C14—H14	120.1
C3—C4—H4	119.8	C13—C14—H14	120.1
C5—C4—H4	119.8	C16—C15—C14	120.37 (15)
C4—C5—C6	119.92 (16)	C16—C15—H15	119.8
C4—C5—H5	120.0	C14—C15—H15	119.8
C6—C5—H5	120.0	C15—C16—C17	120.03 (15)
C5—C6—C1	119.63 (16)	C15—C16—H16	120.0
C5—C6—H6	120.2	C17—C16—H16	120.0
C1—C6—H6	120.2	C16—C17—C18	120.02 (15)
C12—C7—C8	119.59 (15)	C16—C17—H17	120.0
C12—C7—P1	122.65 (12)	C18—C17—H17	120.0
C8—C7—P1	117.57 (12)	C17—C18—C13	120.06 (14)
C9—C8—C7	119.94 (15)	C17—C18—H18	120.0
C9—C8—H8	120.0	C13—C18—H18	120.0
C7—C8—H8	120.0		
			
C7—P1—N1—P1^i^	8.50 (6)	C12—C7—C8—C9	1.0 (2)
C1—P1—N1—P1^i^	129.26 (6)	P1—C7—C8—C9	−174.15 (13)
C13—P1—N1—P1^i^	−115.21 (6)	C7—C8—C9—C10	−0.5 (2)
N1—P1—C1—C2	27.31 (14)	C8—C9—C10—C11	−0.5 (3)
C7—P1—C1—C2	152.11 (12)	C9—C10—C11—C12	1.0 (3)
C13—P1—C1—C2	−90.78 (13)	C10—C11—C12—C7	−0.5 (2)
N1—P1—C1—C6	−156.65 (13)	C8—C7—C12—C11	−0.5 (2)
C7—P1—C1—C6	−31.85 (15)	P1—C7—C12—C11	174.41 (13)
C13—P1—C1—C6	85.26 (14)	N1—P1—C13—C18	3.27 (15)
C6—C1—C2—C3	0.8 (2)	C7—P1—C13—C18	−123.40 (13)
P1—C1—C2—C3	176.89 (12)	C1—P1—C13—C18	120.50 (13)
C1—C2—C3—C4	−0.8 (2)	N1—P1—C13—C14	−173.36 (13)
C2—C3—C4—C5	0.4 (3)	C7—P1—C13—C14	59.97 (14)
C3—C4—C5—C6	0.2 (3)	C1—P1—C13—C14	−56.13 (14)
C4—C5—C6—C1	−0.2 (2)	C18—C13—C14—C15	−0.6 (2)
C2—C1—C6—C5	−0.3 (2)	P1—C13—C14—C15	176.07 (12)
P1—C1—C6—C5	−176.29 (12)	C13—C14—C15—C16	0.0 (2)
N1—P1—C7—C12	−118.52 (12)	C14—C15—C16—C17	0.4 (3)
C1—P1—C7—C12	120.31 (13)	C15—C16—C17—C18	−0.4 (2)
C13—P1—C7—C12	5.46 (15)	C16—C17—C18—C13	−0.1 (2)
N1—P1—C7—C8	56.51 (13)	C14—C13—C18—C17	0.6 (2)
C1—P1—C7—C8	−64.66 (13)	P1—C13—C18—C17	−176.05 (12)
C13—P1—C7—C8	−179.52 (12)		

Symmetry codes: (i) −*x*+1, *y*, −*z*+1/2.

### Hydrogen-bond geometry (Å, °)

**Table:**

*D*—H···*A*	*D*—H	H···*A*	*D*···*A*	*D*—H···*A*
Cl1—H1···Cl1^i^	1.56 (1)	1.56 (1)	3.1045 (9)	173 (3)

Symmetry codes: (i) −*x*+1, *y*, −*z*+1/2.
